# Functional characterization of two melanocortin (MC) receptors in lamprey showing orthology to the MC1 and MC4 receptor subtypes

**DOI:** 10.1186/1471-2148-7-101

**Published:** 2007-06-29

**Authors:** Tatjana Haitina, Janis Klovins, Akiyoshi Takahashi, Maja Löwgren, Aneta Ringholm, Johan Enberg, Hiroshi Kawauchi, Earl T Larson, Robert Fredriksson, Helgi B Schiöth

**Affiliations:** 1 Department of Neuroscience, Unit of Pharmacology, Uppsala University, BMC, Uppsala SE 75124, Sweden; 2 Biomedical Research and Study Centre, University of Latvia, Riga LV 1069, Latvia; 3 School of Fisheries Sciences, Kitasato University, Sanriku, Ofunato, Iwate 022-0101, Japan; 4 Department of Biology, Northeastern University, Boston, MA 02115, USA

## Abstract

**Background:**

The melanocortin (MC) receptors have a key role in regulating body weight and pigmentation. They belong to the rhodopsin family of G protein-coupled receptors (GPCRs). The purpose of this study was to identify ancestral MC receptors in agnathan, river lamprey.

**Results:**

We report cloning of two MC receptors from river lamprey. The lamprey receptors, designated MCa and MCb, showed orthology to the MC1 and MC4 receptor subtypes, respectively. The molecular clock analysis suggested that lamprey MC receptor genes were not duplicated recently and diverged from each other more than 400 MYR ago. Expression and pharmacological characterization showed that the lamprey MCa receptor was able to bind and be activated by both lamprey and human MSH peptides. The lamprey MCa receptor had relatively high affinity for ACTH derived peptides similarly to the fish MC receptors. We found that both of the lamprey MC receptors were expressed in skin, while the MCb receptor was also found in liver, heart and skeletal muscle.

**Conclusion:**

This study shows presence of MC receptors in agnathans indicating early signs of specific functions of melanocortin receptor subtypes.

## Background

G protein-coupled receptors (GPCRs) are the largest superfamily of membrane proteins and serve as targets for more than 30% of all modern drugs. The rhodopsin family is the largest within the GPCR superfamily and includes the α-, β-, γ- and δ-subgroups [1]. The α group of rhodopsin GPCRs includes melanocortin (MC) receptors. The ligands for the MC receptors are the melanocortic peptides: α-, β-, γ-melanocyte stimulating hormones (MSH) and adrenocorticotropic hormone (ACTH), all derived from the precursor proopiomelanocortin (POMC). In addition the MC receptors bind the agouti signalling peptide (ASIP) and agouti related protein (AGPR) that are endogenous antagonists that regulate pigmentation and food intake through the MC1 and MC4 receptors, respectively. The MC receptors are found in five subtypes in mammals and birds, named MC1-MC5 receptors [2,3]. The MC receptor subtypes have very different physiological roles but the MC4 receptor has received extraordinary attention, as it is the most recognized monogenic cause for obesity [4]. MC4 receptor agonists reduce food intake [5] and MC4 receptor antagonists are very efficient in increasing food intake and body weight [6]. The MC4 receptor and the MC3 receptor, which is also involved in regulating energy homeostasis, are some of the most pursued drug targets among GPCRs [7].

We have previously demonstrated the presence of MC receptors in teleost fishes [8,9] and that the MC4 receptor plays an important role for regulation of food intake in teleosts [10,11]. We have also shown that the MC4 receptor is likely to regulate food intake in birds [12]. The zebrafish has six MC receptors due to an additional copy of the MC5 receptor, which we suggest was created through a tetraploidization within the teleost lineage. The teleost Fugu has only four MC receptors as it is lacking the MC3 receptor [8]. Moreover, there are some differences in the ligand preference between teleosts and mammals: ACTH seems to play a more prominent role for the MC receptor in Fugu and trout as compared with α-MSH [13], which is selective to four MSH binding MC receptors in mammals. Furthermore, we recently cloned and characterised MC3, MC4 and MC5 receptors from another gnathostome, the spiny dogfish. The results showed high conservation in sequence and pharmacology compared to human orthologs [14,15], but it is still not known if cartilaginous fishes have any other types of MC receptors. It also remains unknown if MC receptors can be found in more ancient species.

The only extant jawless vertebrates are lamprey and hagfish that form unique basal groups in the craniate lineage intermediate between Amphioxius (protochordate) and gnathostomes (jawed vertebrates). It is proposed that lineages leading to lamprey and hagfish underwent one large-scale genome duplication, whereas the second doubling may have occurred in the gnathostome ancestor lineage. The agnathans differ from other vertebrates by lacking bone tissue and a biting apparatus. They have been suggested to have diverged from the lineage leading to mammals 564 Myr ago [16,17] and represent some of the key species whose genomes have not been sequenced. The MSH ligands are all derived from POMC, which has been intensively studied in lower vertebrates and it seems that the POMC gene has arisen early in chordate evolution [18]. Among the POMC genes that have been cloned, the two POMC genes from the agnathan lamprey are the ones most distantly related to mammals. Degenerative sequences for melanotropins (MSH) and corticotropin (ACTH) peptides were found to be encoded by two distinct genes [19].

In this paper we report the cloning of two MC receptors from river lamprey and a fragment of MC receptor from hagfish. These receptors are the most ancient MC receptors found to date. We expressed one of the lamprey receptors and performed thorough characterization of its pharmacological properties with respect to both human and lamprey melanocortin peptides. Moreover, we show the anatomical distribution of the two lamprey receptors.

## Results

### Cloning of melanocortin receptors from hagfish

The degenerate PCR using genomic DNA from Atlantic hagfish resulted in a fragment of approx 400 bp. The fragment was cloned and sequenced. The sequence was blasted against the GenBank database, which indicated high similarity to MC receptors. The clone was used for high stringency screening of Atlantic hagfish genomic library in lambda GEM-11. The screening did however not result in any positive full-length clones from this library.

### Cloning of melanocortin receptors from river lamprey

The degenerate PCR on genomic DNA of river lamprey resulted in two fragments of approx 400 bp. The fragments were cloned and sequenced. Two different sequences were blasted against the GenBank database and showed similarity to MC receptors. The clones were used for high stringency screening of a river lamprey genomic library. Two positive cosmid clones were isolated and sequenced. Both clones were found to contain the same intronless gene of 1023 bp, corresponding to a putative MC receptor of 341 amino acids. The sequences of both clones were found to be identical to only one of the MC receptor fragments obtained from the PCR. Repeated screening with another fragment as a probe did not result in any other cosmid clone from this library.

In order to obtain the entire coding region of the second MC gene, we performed inverse PCR on lamprey genomic DNA, as previously described [14]. We were able to extend the sequence in both 3' and 5' directions and overlap the stop codon, but not the initiating ATG codon. The genome sequencing reads of sea lamprey, *Petromyzon marinus *were downloaded from [20]. The database was formatted with Formatdb [21]. We searched for MC receptor like sequences in the formatted database using Blastall. The sequences with highest similarity score were used to design primers in the proposed N terminal regional of the second lamprey receptor. The full-length gene was amplified from river lamprey genomic DNA using Pfx polymerase. The fragment was purified and sequenced. The full-length intronless sequence of 1008 bp, corresponding to a putative lamprey receptor of 336 amino acids was obtained.

**Figure 1:**
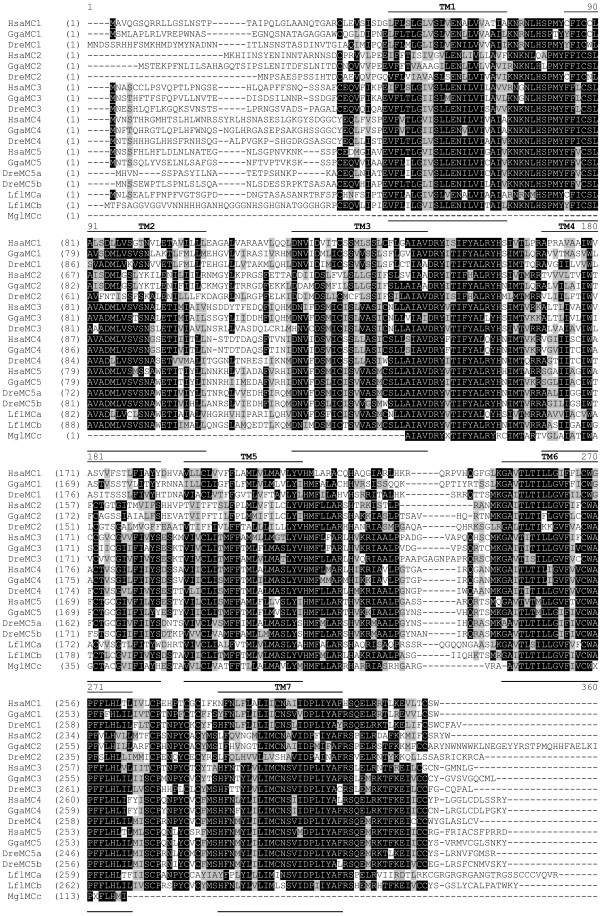
Amino acid sequence alignment of MC receptors constructed using ClustalW 1.8. Putative transmembrane (TM) regions are marked with lines. Black and grey boxes mark conserved and similar amino acid positions, respectively. The abbreviations used: Hsa, human; Gga, chicken; Dre, zebrafish; Lfl, lamprey and Mgl, hagfish. Mgl MCc sequence is partial (119 aa). The accession numbers are listed in "Methods".

The amino acid sequence alignment of the new river lamprey genes and the partial sequence from hagfish together with human, chicken and zebrafish MC receptors is displayed in Figure 1. The percent identities of amino acid sequences are shown in Additional file 1. The lamprey receptor of 341 amino acids exhibits the highest identity, 50–55%, to the MC4 and MC5 receptor subtypes and 48–55% to the MC3 receptors, while the identity to the MC1 receptors is lower, 46–51%. This lamprey receptor has the lowest similarity to the MC2 subtype, 35–43%. This river lamprey receptor was designated MCa receptor. The lamprey receptor of 336 amino acids showed the highest identity of 60–67% to the MC4 and MC5 receptor subtypes and 58–62% to the MC3 subtype, while the similarity to the MC1 and MC2 subtypes was lower, 46–55% and 44–47% respectively. This river lamprey receptor was named MCb receptor. The similarity between the MCa and MCb receptors was 52%. The partial sequence from hagfish showed the highest similarity to MC4 and MC5 subtypes, 56–63% and 59–63%, respectively. The similarity to MC3 subtype was lower, 50–59%, followed by MC1 subtype (50–53%) and MC2 subtype (44–50%). The similarity between hagfish fragment, named MCc receptor, and the corresponding sequence of lamprey MCa and MCb receptors was 54 and 65%, respectively.

### Phylogenetic analysis

**Figure 2:**
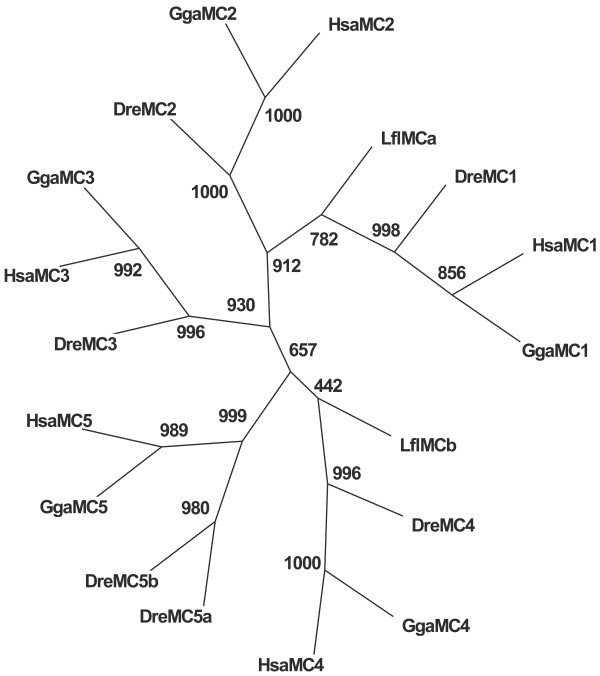
Phylogenetic analysis of full-length amino acid sequences of MC receptors from human, chicken, zebrafish and lamprey. The consensus tree was generated by Neighbor-Joining analysis (Phylip 3.6a3). The numbers above the nodes indicate bootstrap replicates. The abbreviations used: Hsa, human; Gga, chicken; Dre, zebrafish and Lfl, lamprey. The accession numbers are listed in "Methods".

Phylogenetic analysis was performed using Neighbor-joining method and a tree of the MC receptors is presented in Figure 2 and Additional file 2. The topology of the trees was the same with or without out-group (data not shown). In both trees, the lamprey MCa receptor branched at the base of the MC1 receptor group with bootstrap values 782 and 820. In both trees the lamprey MCb receptor branched at the base of the MC4 receptor group with bootstrap values of 442 and 787. The partial hagfish MCc receptor branched at the base of the MC1 and MC2 receptor groups (data not shown).

### Molecular clock analysis

**Figure 3:**
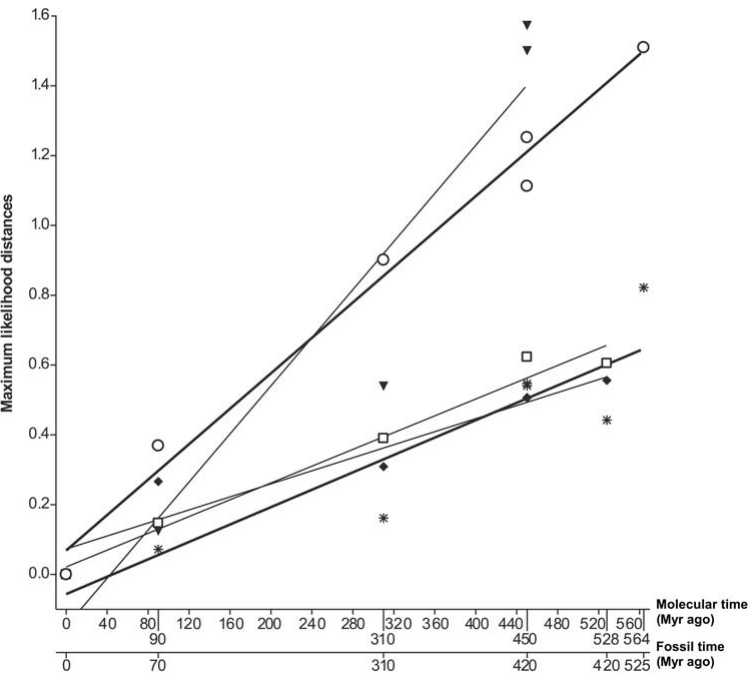
The molecular clock analysis of MC1 (open circle), MC2 (filled triangle), MC3 (open square), MC4 (asterisk) and MC5 (filled diamond) receptor subtypes. Full-length amino acid sequences were used for the calculations. The distances were calculated with Tree-Puzzle v 5.2. Lamprey MCa (open circle) and MCb (asterisk) are plotted at 564 Myr ago. We used the following divergence times from human: mouse – 90(70), chicken – 310(310), zebrafish – 450(420), dogfish – 528 (420) and lamprey – 564 (525) Myr ago. The corresponding calculated distances were plotted against molecular and fossil data for each receptor subtype separately. The lines are linear regression lines.

The theoretical time of divergence of agnathans is estimated 564 MYR for molecular and 525 MYR for fossil data [16]. The MC receptor subtypes displayed different sequence divergence rates as is illustrated in Figure 3. The evolutionary rate of lamprey MCa receptor was most similar to the MC1 receptor subtype. Considering the MC3/4/5 receptor cluster, the lamprey MCb receptor had the divergence rate most similar to the MC4 receptor subtype. This analysis indicated that the MCa and MCb receptors diverged from each other more than 400 MYR. Due to the possible differences in evolutionary rates, this analysis is not conclusive regarding whether the divergence occurred before or after the lamprey split from the lineage leading to mammals.

### Competition and saturation binding experiments on lamprey MC receptor

**Figure 4:**
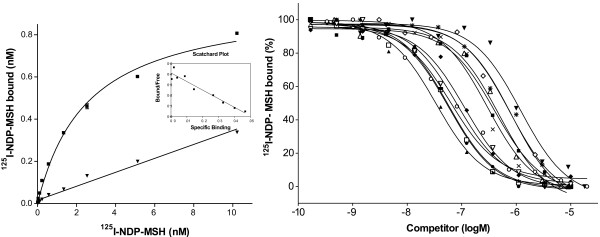
Saturation curves with Scatchard plot and competition curves for the lamprey MCa receptors expressed in HEK-293 EBNA cells. The saturation curves (left) were obtained with ^125^I-labelled NDP-MSH and the figure shows total binding (filled square) and binding in the presence of 2 μM cold NDP-MSH (filled triangle). The lines represent the computer-modelled best fit of the data assuming that ligands bound to one site. The competition curves (right) for NDP-MSH (filled triangle, pointing up), α-MSH (filled square), β-MSH (open circle), γ_1_-MSH (open diamond), ACTH(1–17) (open square), ACTH(1–24) (filled circle), ACTH(1–39) (x), MTII (filled diamond), HS024 (open triangle, pointing down), MSH-A (filled triangle, pointing down), MSH-B (open triangle, pointing up) and ACTH (1–31) (asterisk) were obtained by using a fixed concentration of approx. 0.6 nM ^125^I-labelled NDP-MSH and varying concentrations of the non-labelled competing peptide.

The lamprey MCa receptors were expressed in the HEK-293 EBNA cell line and tested for binding human and lamprey melanocortin peptides. Figure 4 presents saturation and competition curves for the lamprey MCa receptors and Table 1 displays K_d _and K_i _values obtained from saturation and competition experiments, respectively. For comparison, the table also includes previously published results for the human MC receptors tested with the same methodological approach. The results demonstrate that the ligands bind to a single saturable site in the lamprey receptors. The endogenous human ligands α-MSH (p < 0.01), β-MSH, γ-MSH (p < 0.01), ACTH(1–39) (p < 0.01) and truncated ACTH (1–17), ACTH(1–24) had clearly lower affinities for the lamprey MCa receptor, compared to the human MC1 and MC3 receptors, while the same ligands had clearly higher affinities for the lamprey MCa receptor, compared to the human MC4 and MC5 receptors. The synthetic ligands MTII and HS024, however, bound the lamprey MCa receptor with clearly lower affinities than the human MC1, MC3, MC4 and MC5 receptors. The affinity of lamprey MCa receptor to the lamprey peptides was not higher than for the human peptides. The MSH-B had the highest affinity of 352 nM, followed by ACTH(1–31) with 497 nM and the MSH-A with the lowest affinity of 1.50 μM (p < 0.01).

**Table 1:** *K*_*d *_and *K*_*i *_values (mean ± SEM) obtained from saturation and competition curves, respectively, for human and lamprey melanocortin peptide analogues binding lamprey MCa receptor and human MC1, MC3, MC4 and MC5 receptors. * P < 0.01

	**lamprey MCa**	human MC1^a^	human MC3^e^	human MC4^e^	human MC5^b^
Ligand	(nmol·L-1)	(nmol·L-1)	(nmol·L-1)	(nmol·L-1)	(nmol·L-1)
[^125^I] NDP-MSH (*K*_*d*_)	2.46 ± 0.68	0.109 ± 0.062	0.412 ± 0.121	1.78 ± 0.36	5.05 ± 1.00
NDP-MSH (*K*_*i*_)	17.8 ± 9.9	0.046 ± 0.011	0.319 ± 0.064	1.96 ± 0.39	2.39 ± 0.10
α-MSH (*K*_*i*_)	248 ± 59*	0.210 ± 0.089	21.2 ± 5.3	522 ± 122	8240 ± 1670
β-MSH (*K*_*i*_)	54.7 ± 5.1	2.53 ± 0.93	15.1 ± 3.4	387 ± 208	14400 ± 1670
γ_1_-MSH (*K*_*i*_)	684 ± 7*	2.68 ± 0.35^b^	7.45 ± 2.55	51800 ± 12000	42600 ± 6600
ACTH (1–17) (*K*_*i*_)	51.1 ± 3.2	0.230 ± 0.061^c^	14.0 ± 4.5^c^	419 ± 62^c^	4240 ± 1200^c^
ACTH (1–24) (*K*_*i*_)	56.4 ± 10.8	0.209 ± 0.052^c^	32.8 ± 6.7	755 ± 151	2760 ± 780^c^
ACTH (1–39) (*K*_*i*_)	291 ± 20*	3.95 ± 0.67^c^	135 ± 22^c^	2170 ± 120^c^	4920 ± 610^c^
MTII (*K*_*i*_)	71.0 ± 3.3	0.686 ± 0.109^d^	52.6 ± 7.9	6.60 ± 0.82^f^	46.1 ± 7.9^d^
HS024 (*K*_*i*_)	72.5 ± 1.4	18.6 ± 3.3^d^	15.1 ± 3.0	0.341 ± 0.089	3.29 ± 1.15^d^
MSH-A (*K*_*i*_)	1505 ± 262*				
MSH-B (*K*_*i*_)	352 ± 34				
ACTH(1–31) (*K*_*i*_)	497 ± 33				

Data taken from ^a ^[38]; ^b ^[39]; ^c ^[40]; ^d ^[41]; ^e ^[42]; ^f ^[43]

### Measurement of intracellular cAMP in HEK-293 EBNA cells expressing lamprey MC receptor

**Figure 5:**
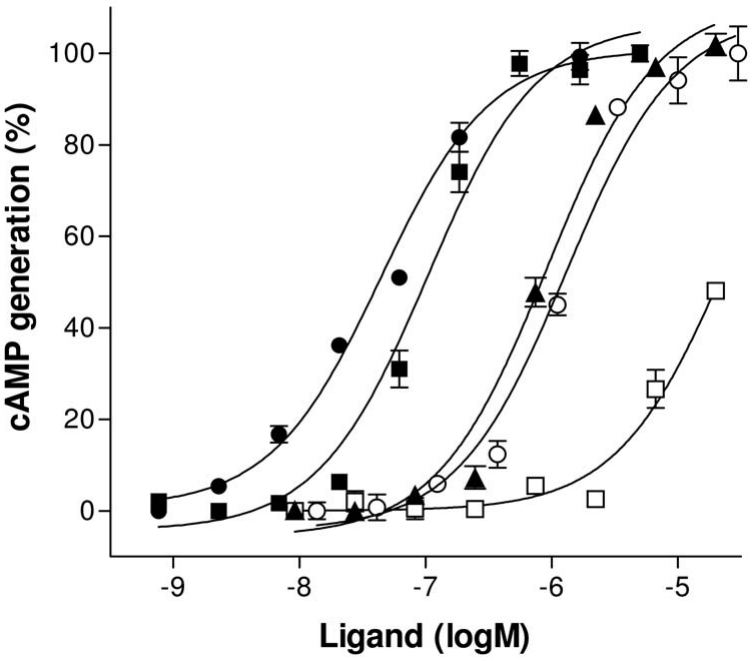
Generation of cAMP in response to α-MSH (filled circle), ACTH(1–24) (filled square), MSH-A (open circle), MSH-B (filled triangle) and ACTH (1–31) (open square) for the lamprey MCa receptors expressed in HEK-293 EBNA cells. Untransfected cells showed no adenylate cyclase activity in response to ligands (data not shown). The cAMP assay was performed in duplicate and repeated two times with each ligand.

The lamprey MCa receptors were expressed in HEK-293 EBNA cells and tested for ability to couple to G-protein and induce the accumulation of cAMP. Human α-MSH, ACTH (1–24) and lamprey MSH-A, MSH-B and ACTH (1–31) were used as ligands. The results are presented in Figure 5. All of the ligands were able to stimulate the accumulation of cAMP. The EC50 value for α-MSH was 49.1 nmol/L, while the ACTH(1–24) displayed lower affinity of 172 nmol/L. The lamprey peptides had higher EC50 values than human peptides: 913 nmol/L for MSH-B (p < 0.01) and 1304 nmol/L for MSH-A (p < 0.01). Stimulation with ACTH(1–31) did not result in a full response.

### Tissue distribution of lamprey MC receptors

**Figure 6:**
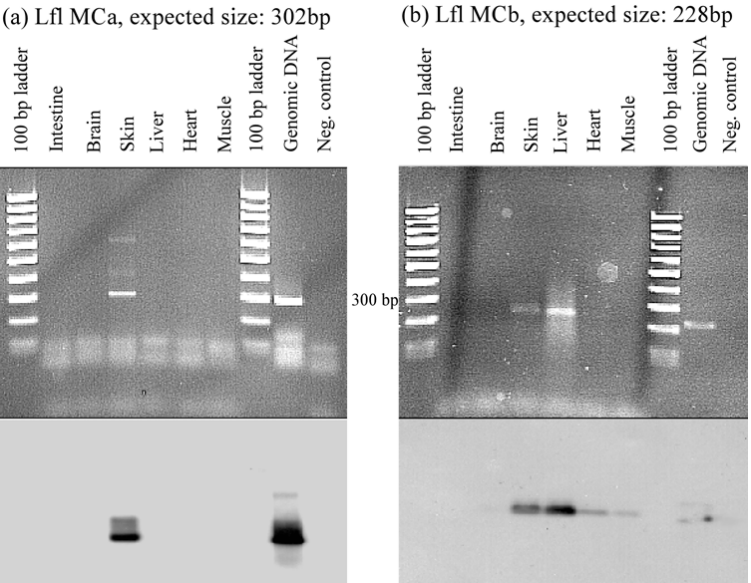
Expression of lamprey MCa (a) and MCb (b) receptor mRNA as determined by RT-PCR. The tissues, controls and expected sizes of the PCR products are denoted at the top of each figure. Ethidium bromide stained agarose gels are presented on top and autoradiographs of Southern blots, hybridised with gene specific probes, are shown below. The PCR and the hybridization were performed three times with qualitatively similar results.

The tissue distribution of the lamprey was determined by RT-PCR. The quality of the mRNA was tested with RT-PCR using β-actin primers and is demonstrated in Additional file 3. The results for both lamprey receptors are shown in Figure 6. Each pair of PCR primers was designed to be specific for only one of the receptor subtypes. The specificity of the PCR reactions was estimated by hybridization blot with the lamprey MC receptor subtype specific probes. There was no cross-hybridization detected between samples (data not shown). A strong signal for the lamprey MCa receptor was detected in the skin. Other tissues did not show the presence of the MCa receptor. Unlike the MCa receptor, the MCb receptor was detected in several tissues. The strongest signal appeared in the liver, while slightly weaker signal was detected in the skin, and finally, two weak signals, only detectable on the Southern blot were found in the heart and muscle of the lamprey. Neither MCa, nor MCb receptors were detected in the brain of the river lamprey.

## Discussion

For the first time, we show the presence of MC receptors in agnathans, both the river lamprey and the Atlantic hagfish. This finding is important as the agnathans are among the most ancient living vertebrates. The phylogenetic and molecular clock analysis indicated that lamprey MCa receptor belongs to the MC1 receptor subtype, while lamprey MCb receptor belongs to the MC4 receptor subtype (see Figure 2 and Additional file 2). However, the amino acid identity analysis indicated that both receptors are most similar to the MC4 and MC5 receptors.

It is well known that different proteins evolve at different rates because of natural selection for functions [16]. Our molecular clock analysis highlights the differences in the sequence divergence rates between the MC1-2 receptor subtypes, that seem to evolve fast, and the MC3-4-5 subtypes that evolve slower (see Figure 3). This is confirmed by amino acid analysis of the different subtypes that for example show much higher similarity to the MC3-4-5 receptors in mammals and fish as compared with the MC1 and MC2 receptors (see Additional file 1). While the MCb receptor clearly seems to belong to the rather slowly evolving MC3-4-5 cluster that surely must have existed prior to the split of agnathans from the lineage of chordates, the MC1 receptor character of the MCa receptor may suggest that an ancestor to the MC1 receptor was also present at this stage in evolution. It is interesting to note that the percentage identity between the MCa and MCb receptors is much lower than for example the dogfish MC4 or MC5 receptors, which are the evolutionary most closely related MC receptors identified so far. It seems thus clear that these two lamprey receptor genes were not duplicated recently. The molecular clock analysis supports this, placing the divergence of these two genes more than 400 MYR ago. None of the ancestral MC receptors displayed any high similarity to the MC2 (ACTH) receptor subtype. This could mean that either there exists an additional subtype in lamprey with MC2 receptor characteristics or that the MC2 receptor appeared at a later stage. We could not identify any MC receptors in invertebrates or in the tunicate Ciona, despite our very comprehensive searches with HMMs, which has in our hands proven to be a very effective tool to find divergent sequences [22,23]. Moreover, our initial searches indicate that the cephalochordate amphioxus does not have any MC receptors either (data not shown). This suggests that the MC receptor family appeared first in the agnathan lineage, after the divergence of craniates (vertebrates), but before the appearance of jawed vertebrates. Taken altogether, the genes we isolated and characterised here, could code for the most ancestral MC receptors.

It has previously been reported that orthologs to the large precursor peptide, proopiomelanocortin (POMC) are found in lamprey and that these are encoded by two genes, POM (proopiomelanotropin) and POC (proopiocortin). POM encodes β-endorphin and two degenerated sequences termed MSH-A, MSH-B, while POC encodes another β-endorphin and an ACTH like sequence [19]. The position of MSH-B corresponds to that of ACTH, while the position of MSH-A corresponds to that of β-MSH in mammalian POMC. The mammalian and fish melanocortin peptides (α-, β- and γ-MSH) cannot be distinguished. Similarity in exon/intron organization, the position of β-endorphin and ACTH/MSH-B suggests that POM and POC appeared from a duplication of an ancestral POMC gene [24]. It seems thus clear that ancestral (prior to the split of lamprey) POMC gene contained orthologs of both ACTH and β-endorphin. The core binding sequence HFRW is conserved between human and sea lamprey melanocortin peptides but α-MSH and ACTH (not β-, γ- or δ-MSH) have much larger conserved motif SYSMEHFRWGKP in all species described so far, except the gecko *Eublepharis macularius*, [25]. For lamprey MSH-A, MSH-B and ACTH, this common motif looks like xYxMxHFRWGxP [26]. It was therefore interesting to test if these four amino acid substitutions would affect the pharmacology. Because we had ancestor-like MC receptor clone from river lamprey, we decided to synthesize the melanocortin peptides from lamprey and test their pharmacological properties with this receptor. We showed that the lamprey MCa receptor was able to bind both lamprey and human endogenous MSH peptides as well as their synthetic analogues. This could be related to the fact that the core binding sequence of the MSH peptides (HFRW) is well conserved in the lamprey. The ability of the lamprey receptor to bind all the MC ligands that we tested shows that the lamprey MCa receptor is indeed a MSH binding receptor and that it does not have the MC2 (ACTH) receptor subtype characteristics to bind only ACTH, but not MSH peptides. The binding profile of this lamprey receptor is not in particular similar to the MC1 receptors, having much lower affinities for all MC receptor ligands. It did neither have the characteristics of the mammalian MC4 receptor, which has preference to β-MSH and low affinity to γ-MSH, or the mammalian MC3 receptor, which has preference to γ-MSH. Despite the generally low affinity of the MCa receptor it still binds all human ligands with higher affinities than the human MC5 receptor. The potency order of the lamprey MCa receptor was most similar to the dogfish MC3 receptor [14], while affinity levels in general were most similar to the trout MC4 and MC5 receptors [27]. Interestingly, the MCa receptor shows higher affinity for γ-MSH and ACTH than the human MC4 and MC5 receptors. All this points to the direction that the ancestral MC receptors had a rather promiscuous binding profile and that the specification of the different subtypes has occurred at a later stage in the evolution. The lamprey receptor had highest affinity for the ACTH(1–17). We have previously reported that MC receptors from non-mammalian vertebrates seem to prefer ACTH peptides before other ligands [13,14,27]. This pharmacology of the lamprey receptor further supports our previous hypothesis that ACTH peptides could have acted as the "original" ligand at the MC receptors. It is also notable that the lamprey receptor has higher affinity to the lamprey ACTH peptide and the relatively similar MSH-B peptide, as compared with MSH-A, which also support this hypothesis. The low affinity of MSH-A may indicate that there is another MC receptor in lamprey that has this peptide as ligand, and that could obviously be the MCb receptor. Moreover, we demonstrated the ability of the lamprey MCa receptor to functionally couple to the Gs pathway in response to human and lamprey endogenous ligands. This is in line with all previously characterised MC receptors further adding evidence to the notion that the coupling preferences of GPCRs are in general very well conserved through the evolution.

We found that both of the lamprey MCa and MCb receptors are expressed in the skin, while the MCb receptor was also found in liver, heart and skeletal muscle. The skin is one of the major sites of expression for the mammalian MC1 receptor and the role of ACTH peptides for skin pigmentation has been highlighted in several studies [28]. Our new results on the lamprey receptors may indicate that the skin is one of the original sites of expression for the MC receptors. The fact that this receptor binds ACTH with relatively high affinity could fit with a conserved role of ACTH in pigmentation. Like the MCb receptor, MC5 is expressed in liver, heart and skeletal muscle in mammals [29]. These findings lend further support to similarities of the MCa receptor with the MC1 receptor and the MCb receptor with the MC3-4 and -5 receptors. The POM and POC genes we previously found to be expressed in pars intermedia and pars distalis, respectively, of lamprey pituitary [26]. Neither of the lamprey MC receptors was detected in the brain. This could suggest that the melanocortin system functioned primarily as endocrine hormonal system where the melanocortin peptide hormones were released centrally to reach the receptors in peripheral tissues such as the skin. This is in line with how ACTH works for the adrenal MC2 (ACTH) receptors while it is in stark contrast to how POMC products work for the other receptors where they act as local hormones in the brain (MC3 and MC4) and the skin (MC1). This could suggest the melanocortin system acted originally in an endocrine hormonal fashion while the function to act as a local hormonal system evolved later. Our previous findings in several fish species such as the spiny dogfish, Fugu and zebrafish show important central expression pattern for the MC receptors. This could suggest that the central functions for MC receptors developed after the split of agnathans but prior to the split of gnathostomes. It is also possible that the central functions were developed more early on, but that these may have been lost in the lineage leading to lamprey that could be related to parasitic living conditions of these species. Moreover, it is also possible that there exist additional lamprey MC receptors with central functions, but that we were unable to isolate them. Recent evolutionary theories suggest that duplicate genes more commonly survive by function partitioning or subfunctionalization, rather than acquiring new functions [30]. The unique location of the MCa receptor in the skin and wide distribution of the MCb receptor in skin, liver, heart could indicate subfunctionalization. It is possible that the MCa receptor took over the function in the skin from the wide range of functions of ancestral gene and that the specialisation, which is so apparent for the mammalian MC receptor subtypes, took place later for the MCb receptor subtype.

## Conclusion

In summary, we have described MC receptors in the most ancient living vertebrate, the river lamprey. We have characterized these ancestral receptors by phylogeny, evolutionary divergence rate, pharmacology and anatomical distribution. We show that a lamprey MC receptor can bind MSH peptides and like non-mammalian vertebrates, have preference to human ACTH peptides before other ligands. The anatomical characterization indicates that the MCa receptor is a skin receptor, while the MCb receptor has a more broad distribution. The duplication of MC subtypes took place most likely before the divergence of jawed vertebrates or gnathostomes.

## Methods

### Cloning and sequencing of melanocortin receptor fragments from river lamprey and Atlantic hagfish

Degenerate PCR primers were designed from sequence alignment of all known MC receptors to match highly conserved receptor regions. Several primer pairs were constructed and two pairs turned out to be effective for amplification of partial sequences from jawless vertebrates: 5'-GGCCATCGCCGTCGAYMGNTAYRT-3', 5'-GATCATGTGCAGGAAGAMNGGNVHCCA-3'; and 5'-TAYKTNACIATHTTYTRYGC-3', 5'-AANGCRAADATIARNGGRTG-3' (I represents inosine, degenerate symbols are IUBMB codes). Genomic DNA was extracted from liver of river lamprey and hagfish. About 500 mg of tissue was homogenized in lysis buffer containing 100 mM EDTA, 10 mM Tris and 1% SDS and centrifuged for 5 min at 11500 g. The supernatant was extracted with saturated phenol, phenol/chloroform/isoamyl alcohol (25:24:1) and chloroform. The DNA was precipitated with 1 volume of isopropanol in the presence of 1 M NH_4_Ac (10 v/1 v) and washed with 70% ethanol. Touch-down PCR was performed with Taq polymerase (Fermentas, Lithuania) under the following conditions: initial denaturation for 5 min at 95 C, followed by 21 cycle of 30 s at 94 C, 45 s at 52-42 C (lowered by 0.5 C per cycle) and 90 s at 72 C. This was followed by 25 cycles of 30 s at 94 C, 40 s at 50 C and 1 min at 72 C, with a final extension of 5 min at 72 C.

The PCR products were separated on a 1.5% agarose gel, and the products of desired length were cut out from the gel, purified using the Gel Extraction Kit (Qiagen, Sweden) and cloned into the plasmid vector pCRII from TOPO cloning kit and transformed into DH5α cells (Invitrogen, Sweden) according to the manufacturers' instructions. The plasmid inserts were sequenced with vector-specific primers using ABI PRISM Big Dye Terminator Cycle Sequencing Ready Reaction Kit v2.0 and analyzed by an automated ABI PRISM 310 or ABI PRISM 3100 fluorescent-dye sequencer (Applied Biosystems, Sweden). Sequences were analysed with Seqman software from DNAstar package (Lasergene, USA). Sequences were compared against database assemblies with BLASTN and BLASTX [31].

### Screening of river lamprey genomic library and isolation of full-length genes

Filters from a river lamprey cosmid library in vector EMBL3 containing approximately 700 000 clones (roughly three genome equivalents) were obtained from RZPD (Deutsches Ressourcenzentrum für Genomforschung GmbH, Germany). MC receptor-like fragments were labelled with ^32^P using Megaprime Labelling System (Amersham Biosciences, Sweden) and used as probes. Hybridization was performed at 65°C in 25% formamide (Merck Eurolab AB, Sweden), 6× SSC, 10% dextran sulfate (Amersham Biosciences), 5× Denhardt's solution, and 0.1% SDS overnight. The filters were washed five times in 0.2 × SSC and 0.1% SDS at 65°C for 1 h. After exposure to autoradiographic films, two individual cosmid clones were selected for further characterization. The cosmid cultures were grown in Luria-Bertani (LB) medium in presence of 20 μg/ml kanamycin. Cosmid DNA was isolated with the Large-Construct kit (Qiagen) according to manufacturer's instructions. Already known MC receptor sequence fragments were used to design sequencing primers. Isolated DNA was sequenced with these primers and obtained sequence was used to design additional sequencing primers. This strategy was applied until whole MC receptor gene sequences were obtained. To confirm their similarity to MC receptor genes, sequences were compared against database assemblies with BLASTN and BLASTX [31].

### Screening of Atlantic hagfish genomic library

Atlantic hagfish lambda GEM-11 genomic library (Promega, USA) was plated with bacteria host strain. Approximately, 600 000 plaques were lifted with MSI nylon transfer membranes (Osmonics Inc., USA) in duplicates and filters were hybridized with ^32^P-labeled probe as described above. Filters were exposed to autoradiographic film (Amersham Biosciences) and analyzed.

### Alignment and phylogenetic analysis of cloned melanocortin receptors

Alignment of the predicted full-length amino acid sequences for the lamprey MCa (341aa) and MCb (336aa) receptors together with other known MC receptors was generated with ClustalW 1.8 software [32]. The following receptor sequences (with their accession codes) were retrieved from GenBank for this analysis: human, *Homo sapiens*, Hsa MC1 [GenBank:NM_002386], MC2 [GenBank:NM_000529], MC3 [GenBank:XM_009545], MC4 [GenBank:NM_005912], MC5 [GenBank:XM_008685]; mouse, *Mus musculus*, Mmu MC1 [GenBank:NM_008559], MC2 [GenBank:NM_008560], MC3 [GenBank:NM_008561], MC4 [GenBank:NM_016977], MC5 [GenBank:NM_013596]; chicken, *Gallus gallus*, Gga MC1 [GenBank:D78272], MC2 [GenBank:AB009605], MC3 [GenBank:AB017137], MC4 [GenBank:AB012211], MC5 [GenBank:AB012868], Fugu, *Takifugu rubripes*, Tru MC1 [GenBank:AAO65549], MC2 [GenBank:AAO65550], MC4 [GenBank:AAO65551], MC5 [GenBank:AAO65552], zebrafish, *Danio rerio*, Dre MC1 [GenBank:NM_180970], MC2 [GenBank:NM_180971], MC3 [GenBank:NM_180972], MC4 [GenBank:AY078989], MC5a [GenBank:AY078990] and MC5b [GenBank:AY078991], goldfish, *Carassius auratus*, Cau MC4 [GenBank:CAD58853], MC5 [GenBank:CAE11349]; rainbow trout *Oncorhynchus mykiss*, Omy MC4 [GenBank:AY534915] and OmyMC5 [GenBank:AY534916]; and spiny dogfish, *Squalus acanthias*, SacMC3 [GenBank:AY560605], Sac MC4 [GenBank:AY169401] and SacMC5 [GenBank:AY562212]. The new genes have the following accession numbers: river lamprey, *Lampetra fluviatilis*, LflMCa [GenBank:DQ213059], LflMCb [GenBank:DQ213060] and Atlantic hagfish, *Myxine glutinosa*, MglMCc [GenBank:DQ213061]. The alignment was bootstrapped 1000 times using SEQBOOT from Phylip 3.6a3 package [33] to obtain a total of 1000 different alignments. Protein distances were calculated using PROTDIST from Phylip 3.6 a3. The Jones-Taylor-Thornton matrix was used for the calculation. The trees were calculated from the 1000 different distance matrixes, previously generated with PROTDIST, using NEIGHBOR from Phylip 3.6 a3. As a result 1000 Neighbor-Joining trees were constructed. The bootstrapped consensus Neighbor-Joining tree was obtained with CONSENSE from Phylip 3.6a3 and plotted using TreeView 1.6.6 [34]. The partial amino acid sequence of the hagfish MglMCc (119aa) was aligned with other known MC receptors and the phylogenetic tree was calculated as described above. The position of the hagfish MCc receptor and the bootstrap value were added to the tree containing the lamprey MC receptors.

### Molecular clock analysis

The full length amino acid sequences of all known melanocortin receptors from human, mouse, chicken, zebrafish, dogfish and two sequences from lamprey were aligned with ClustalW. The constructed alignment in Phylip format was used to calculate the Neighbor-Joining tree, as described above. The alignment and the calculated tree were used as an input files for Tree-Puzzle v5.2 program [35]. The maximum likelihood distances with molecular clock assumption were calculated with 10 000 puzzling steps, using JTT matrix [36], and mixed model of rate heterogeneity with 1 invariable and 8 gamma rates, otherwise default settings were applied. The following molecular (fossil) data displaying species divergence from human were applied: human, 0 (0) Myr ago; mouse, 90 (70) Myr ago; chicken, 310 (310) Myr ago; zebrafish, 450 (420) Myr ago; dogfish, 528 (420) Myr ago and lamprey, 564 (525) Myr ago [16]. The corresponding calculated distances, except for lamprey MC receptors, were plotted against the molecular and fossil data for each receptor subtype separately. The linear regression equation was calculated for each receptor subtype. This equation was used to calculate the distances for lamprey MCa and MCb receptors for each receptor subtype. These distances were compared to the tree-puzzle distances for lamprey MCa and MCb receptors. The closest tree-puzzle distances were plotted at the corresponding subtype.

### Cloning into pCEP expression vector

Full-length coding sequences of new receptor genes were amplified by means of PCR from genomic DNA with Pfx polymerase (Invitrogen) using primers containing *Hind*III and *Xho*I restriction sites at the N and C terminus respectively. Obtained fragments were then digested with both restriction enzymes and gel purified prior to ligation into modified pCEP expression vector ([Amp^+^], [Hygro^+^]; CMV promoters; SV40 PolyA) [37]. The construct was sequenced as previously described.

### Expression of receptors

HEK-293 (human embryonic kidney) cells with Epstein-Barr nuclear antigen (EBNA) grown to 50–70% confluence were transfected with 10 μg of the construct using FuGENE-6 Transfection Reagent (Roche, Sweden) according to the manufacturers' instruction. The cells were grown in Dulbeccos Modified Eagle Media and F-12 Nutrient Mixture (D-MEM/F-12) (1:1) with GlutaMAX I containing 10% foetal bovine serum, 100 u/ml penicillin, 100 μg/ml streptomycin, 2.5 ug/ml amphotericin B, and 250 μg/ml geneticin G-418 (Invitrogen); in humidified atmosphere of 95% air and 5% CO_2 _at 37°C. Semi-stable cell lines, expressing target receptor, were obtained by selecting for growth in the presence of 100 μg/ml hygromycin B (Invitrogen), first added 24 hours after transfection.

### The synthesis and purification of lamprey melanocortin peptides MSH-A, MSH-B and ACTH(1–31)

Sea lamprey (*Petromyzon marinus*) MC peptides were synthesized using an automated solid-phase peptide synthesizer (Shimadzu PSSM-8). A series of reactions was performed according to the manufacturer's instruction as follows. ACTH(1–31) was synthesized on a TGS-AC-Fmoc-Asn-resin (30 mg/vessel, capacity 200 μmol/g), MSH-A was synthesized on a TGS-AC-Fmoc-Phe-resin (31 mg/vessel, capacity 210 μmol/g), and MSH-B was synthesized on a TGS-RAM95%TFA-resin (33 mg/vessel, capacity 200 μmol/g). Fmoc-amino acid [60 μmol for ACTH(1–31), 65 μmol for MSH-A, and 66 μmol for MSH-B] was activated with benzotriazol-1-yl-oxy-tris(pyrrolidino) phosphonium hexafluorophophate [60 μmol for ACTH(1–31), 65 μmol for MSH-A, and 66 μmol for MSH-B] in the presence of 1-hydroxybenzotriazole [60 μmol for ACTH(1–31), 65 μmol for MSH-A, and 66 μmol for MSH-B] and *N*-methylmorpholine [90 μmol for ACTH(1–31), 98 μmol for MSH-A, and 99 μmol for MSH-B] in dimethylformamide (DMF) [210 μl for ACTH(1–31), 228 μl for MSH-A, and 231 μl for MSH-B] for 30 min. The manipulations in each cycle consisted of (i) deprotection of the Fmoc group with 30% piperidine/DMF (500 μl, 6 min, 2 times), (ii) washing with DMF (600 μl, 5 times), (iii) coupling of each amino acid in DMF, and (iv) washing with DMF (600 μl, 5 times). After the completion of all amino acid couplings, cleavage of the peptides and deprotection of the side chain were performed in TFA/water/thioanisol/ethylmethyl sulfide/ethanditiol/thiophenol = 165/10/10/6/5/4 (v/v) supplemented with 0.5% 2-methylindole (less than 200 μl) for 6 hr at room temperature. The peptides were precipitated by the addition of prechilled ether (10 ml) and lyophilized from 0.1% TFA. Peptides were purified by rpHPLC on a TSKgel ODS-120T column (0.46 × 25 cm) with a linear gradient (10 to 70%) of acetonitrile containing 0.1% TFA. The amino acid sequences of the synthetic peptides were confirmed by sequence analysis using a protein sequencer PPSQ-21A (Shimadzu, Kyoto, Japan), and mass spectrometry using an AXIMA-CFR plus mass spectrometer (Shimadzu).

### Radioligand binding assay

HEK-293 EBNA cells expressing MC receptors were harvested from plate and resuspended in binding buffer composed of 25 mM HEPES, 2.5 mM CaCl_2_, 1 mM MgCl_2 _and 0.2% bacitracin, pH adjusted to 7.4. To obtain the membranes, cells were mechanically homogenized with Ultra Turrax. Cell suspensions were centrifuged for 3 min at 190 g and membranes were collected from the supernatant by centrifugation for 15 min at 20000 g. The pellet was resuspended in binding buffer. The binding was performed in a final volume of 100 μl for 2 h at room temperature. Saturation experiments were carried out with serial dilutions of [^125^I] NDP-MSH { [Nle^4^, D-Phe^7^]α-MSH}, labelled by the Chloramine-T method. Non-specific binding was determined in the presence of 1 μM unlabeled NDP-MSH. Competition experiments were performed with constant 0.6 nM concentration of [^125^I] NDP-MSH and serial dilutions of competing unlabelled human ligands: NDP-MSH, α-MSH, β-MSH, γ_1_-MSH, ACTH (1–17) ACTH (1–24) ACTH (1–39), MTII, HS024) (Neosystem, France) and lamprey ligands: MSH-A, MSH-B, ACTH(1–31). The membranes were collected by filtration on Glass Fibre filters, Filtermat A (Perkin Elmer, USA), using a TOMTEC Mach III cell harvester (Orange, CT, USA). The filters were washed with 5 ml per well of 50 mM Tris HCl (pH 7.4) and dried at 50°C. MeltiLex A scintillator sheets (Perkin Elmer, USA) were melted on dried filters and radioactivity was counted with an automatic Microbeta counter 1450 (Perkin Elmer). Binding assays were performed in duplicate with at least three independent experiments. Non-transfected cells did not show any specific binding with [^125^I] NDP-MSH. The results were analysed with Prism 3.0 software package (GraphPad, USA). Binding affinities were compared with one-way ANOVA Tukey's post-hoc tests.

### cAMP detection assay

Cyclic adenosine monophosphate (cAMP) production was determined on semi-stable HEK-293 EBNA cells expressing the target MC receptors. A confluent layer of cells was incubated for 3 hours with 2.5 μCi/ml of [^3^H] ATP (specific activity 29 Ci/mmol; Amersham Biosciences). Cells were collected, washed and resuspended in buffer containing 137 mM NaCl, 5 mM KCl, 0.44 mM KH_2_PO_4_, 4.2 mM NaHCO_3_, 1.2 mM MgCl_2 _× 6H_2_O, 20 mM HEPES, 1 mM CaCl_2_, 10 mM glucose and 0.5 mM isobutylmethylxanthine (Sigma-Aldrich, Germany), pH adjusted to 7.4. Resuspended cells were incubated at 37°C for 10 min. Stimulation reaction was performed at 37°C for 20 min in a final volume of 150 μl containing approximately 2 × 10^5 ^cells and various concentrations of lamprey MSH-A, lamprey MSH-B, lamprey ACTH(1–31), human α-MSH and human ACTH(1–24). After incubation cells were centrifuged and 200 μl of 0.33 M perchloric acid was added to pellets to lyse the cells. The cells were frozen, thawed and centrifuged. 200 μl of lysate was added to Dowex 50W-X4 resin columns (Bio-Rad), previously washed with 2 × 10 ml H_2_O. As an internal standard, 750 μl 0.33 M perchloric acid containing 0.5 nCi/ml [^14^C] cAMP (Amersham Biosciences) was added to each column. Columns were washed with 2 ml H_2_O to remove ATP, which was collected in scintillation vials to estimate the amount of unconverted [^3^H] ATP. 4 ml of Ready Safe scintillation cocktail (Perkin Elmer) was added to the vials before counting. Dowex columns were then placed over alumina (Sigma-Aldrich) columns (pre-washed with 8 ml 0.1 M imidazole) and the cAMP was transferred onto the alumina column using 10 ml H_2_O. cAMP was eluted from alumina column with 4 ml 0.1 M imidazole and collected into scintillation vials to which 7 ml of scintillation fluid was added. ^3^H and ^14^C were counted on Tri-carb liquid scintillation beta counter. The amount of obtained [^14^C]cAMP was expressed as a fraction of total [^14^C]cAMP ([^14^C]cAMP/total [^14^C]cAMP) and was used to estimate column efficiency in order to standardize [^3^H]cAMP. Results were calculated as the percent of total [^3^H]ATP (obtained as a sum of [^3^H]ATP from first column and [^3^H]cAMP from second column) to [^3^H]cAMP and used to determine EC_50 _values by non-linear regression using Prism 3.0 software. All experiments were performed in duplicate and repeated three times. EC50 values were compared with one-way ANOVA Tukey's post-hoc tests.

### RT-PCR (reverse transcription PCR) and Southern analysis

In order to perform RT-PCR analysis of river lamprey MC receptors total RNA was isolated from brain and a number of peripheral tissues: skin, muscle, heart, liver and intestine of river lamprey. Tissues were rapidly dissected from sacrificed animals within five minutes and immersed in RNAlater (Ambion, USA) for 24–72 hrs at 4°C before RNA extraction. Tissues were homogenized on ice and total RNA was isolated using Rneasy Mini Kit (Qiagen, USA) including processing with DNA shredder and DNAse I treatment (Qiagen) as recommended in manufacturer's protocol. Since some of the samples retained genomic DNA, total RNA was exposed to another treatment with 1 u/μl RNase-free DNaseI (Roche, Sweden) for 10 min, followed by heat inactivation of DNase for 5 min at 70°C. Absence of genomic DNA in RNA preparations was confirmed by PCR with primers specific to β-actin gene using 10–100 ng of total RNA as a template and genomic DNA as a positive control. Messenger RNA was reverse transcribed using the 1^st ^Strand cDNA Synthesis kit (Amersham Biosciences). The produced cDNA was used as a template for PCR with the specific primers for the receptor genes. The RT-PCR primers constructed for Lfl MCa were 5'-CTG CTT CAT CTG CAG CCT GG-3' and 5'-TCA CGA CGC ACG CCG CCC ACA-3' (expected product size 302 bp); primers for Lfl MCb were 5'-AGC ACA AGA CGA GCA TGC GT-3' and 5'-TGT GGC GCA TCT CGT GCG AG-3' (expected product size 228 bp). The PCR was performed with Taq polymerase (Invitrogen) under the following conditions: initial denaturation for 1 min, 20 s at 95°C, 30 s at appropriate annealing temperature, 60 s at 72°C for 30 or 40 cycles and final 5 min at 72°C. The PCR products were analysed on a 1% agarose gel. DNA from the gel was transferred onto nylon filters overnight using 0.4 M NaOH. The filters were hybridized with a random-primed ^32^P-labelled, receptor specific probe. Probes were generated with Megaprime DNA labelling kit RPN1607 (Amersham Biosciences) using sequence verified PCR products amplified from plasmids containing river lamprey MC receptor genes. Hybridization was carried out at 65°C in 25% formamide, 6× SSC, 10% dextran sulfate, 5× Denhardt's solution and 0.1% SDS overnight. The filters were washed three times in 0.2× SSC, 0.1% SDS for 1 h at 65°C and exposed to autoradiography film (Amersham Biosciences). Sequence analysis of the PCR products confirmed the presence of target receptor sequences. The RT-PCR reactions and hybridization were performed at least two times each.

## Abbreviations

ACTH – adrenocorticotropic hormone

AGPR – agouti related protein

ASIP – agouti signalling peptide

GPCR – G protein-coupled receptor

HEK-293 EBNA – human embryonic kidney cells with Epstein-Barr nuclear antigen

HMM – Hidden Markov Model

MC – melanocortin

MSH – melanocyte stimulating hormone

NDP-MSH – [Nle4, D-Phe7]α-MSH

POC – proopiocortin

POM – proopiomelanotropin

POMC – proopiomelanocortin

## Authors' contributions

HBS initiated the project. JK and TH performed most of the cloning and expression analysis. ETL performed a degenerated PCR. AR screened genomic libraries. AT and HK synthesised the lamprey peptides. ML and JE performed most of the pharmacological experiments. RF and TH performed the sequence analysis and phylogenetic analysis. TH and HBS interpreted the results and wrote the paper.

## Supplementary Material

Additional File 1 The percentage identity of the full-length amino acid sequences for the melanocortin receptor subtypes from different species. The abbreviations used: Hsa, human; Mmu, mouse; Gga, chicken; Tru, Fugu; Dre, zebrafish Omy, trout; Cau, goldfish; Sac, dogfish; Lfl, lamprey and Mgl, hagfish. The accession numbers are listed in "Methods". The MglMCc sequence is partial (119 aa). Click here for file

Additional File 2 Phylogenetic analysis of full-length amino acid sequences of lamprey MC receptors together with most closely related human α-group rhodopsin GPCRs. The consensus tree was generated by Neighbor-Joining analysis (Phylip 3.6a3). The numbers above the nodes indicate bootstrap replicates. The abbreviations used: Hsa, human and Lfl, lamprey. The sequences of annotated genes were downloaded from GenBank. Click here for file

Additional File 3 Expression of lamprey β-actin mRNA, determined by RT-PCR. Expression is presented on ethidium bromide stained agarose gel. The tissues and controls are denoted at the top of the figure. The PCR was performed two times with qualitatively similar results. Click here for file
