# Supplementary material for: Functional characterization of two melanocortin (MC) receptors in lamprey showing orthology to the MC1 and MC4 receptor subtypes
Source: BMC Evol Biol. 2007 Jun 29;7:101. doi: 10.1186/1471-2148-7-101 (PMC1925065; doi:10.1186/1471-2148-7-101)
Supplement: Additional File 1 — The percentage identity of the full-length amino acid sequences for the melanocortin receptor subtypes from different species. The abbreviations used: Hsa, human; Mmu, mouse; Gga, chicken; Tru, Fugu; Dre, zebrafish Omy, trout; Cau, goldfish; Sac, dogfish; Lfl, lamprey and Mgl, hagfish. The accession numbers are listed in "Methods". The MglMCc sequence is partial (119 aa). [file 1471-2148-7-101-S1.pdf]

	HsaMC1	MmuMC1	GgaMC1	TruMC1	DreMC1	HsaMC2	MmuMC2	GgaMC2	TruMC2	DreMC2	HsaMC3	MmuMC3	GgaMC3	DreMC3	SacMC3	HsaMC4	MmuMC4	GgaMC4	TruMC4	DreMC4	OmyMC4	CauMC4	SacMC4	HsaMC5	MmuMC5	GgaMC5	TruMC5	DreMC5a	DreMC5b	OmyMC5	CauMC5	SacMC5	LflMCa	LflMCb	MglMCc
HsaMC1		76	60	57	54	40	39	40	42	41	46	45	45	49	47	47	47	47	50	48	48	48	48	44	47	46	47	48	47	47	47	49	50	46	53
MmuMC1			59	58	54	39	38	39	41	40	47	47	47	49	47	47	45	46	49	48	49	48	48	47	47	48	48	48	48	47	48	47	46	48	50
GgaMC1				64	61	42	42	40	41	39	48	46	47	49	47	47	47	48	54	52	50	52	50	49	51	50	49	51	51	51	51	48	51	50	51
TruMC1					78	41	42	41	42	37	50	49	54	51	51	52	51	50	53	53	52	53	53	49	54	52	53	52	53	53	53	53	48	55	53
DreMC1						41	40	40	43	39	47	47	50	48	50	51	50	48	53	51	50	51	51	49	50	51	52	52	51	54	53	53	46	51	52
HsaMC2							89	69	49	49	45	46	47	47	47	47	48	48	49	48	49	48	47	45	46	48	48	46	47	48	46	49	42	45	44
MmuMC2								68	50	49	44	46	48	45	46	46	48	47	47	47	47	47	46	46	44	46	46	44	47	47	45	48	42	46	45
GgaMC2									48	49	44	44	45	45	45	46	46	46	48	47	48	47	48	43	44	47	45	45	44	45	44	47	35	44	45
TruMC2										55	42	40	42	42	42	46	46	47	44	46	46	46	45	44	45	46	46	46	45	45	45	44	43	47	47
DreMC2											44	44	43	42	41	45	46	44	47	46	45	46	44	43	42	44	42	41	43	43	41	43	40	46	50
HsaMC3												88	74	69	67	58	58	57	64	62	60	62	62	60	58	62	59	61	62	60	60	60	55	60	58
MmuMC3													74	69	67	57	58	56	62	61	61	61	61	59	58	62	58	60	61	58	59	61	54	58	56
GgaMC3														68	76	62	61	61	67	65	65	65	65	62	63	65	62	64	64	64	63	66	52	62	58
DreMC3															66	59	59	58	66	64	64	63	66	62	61	62	62	64	64	62	64	64	54	60	59
SacMC3																61	61	62	66	64	66	64	65	61	64	65	63	65	64	64	64	65	48	62	50
HsaMC4																	93	87	68	70	69	69	73	61	61	65	65	64	64	62	63	65	50	61	60
MmuMC4																		86	68	70	69	69	71	62	61	64	64	64	62	62	62	64	51	61	60
GgaMC4																			68	70	70	70	70	61	61	65	64	64	62	63	64	66	51	60	56
TruMC4																				78	80	78	75	63	63	67	67	67	66	68	66	68	54	65	62
DreMC4																					78	97	77	64	64	68	66	69	67	68	68	67	53	66	63
OmyMC4																						78	78	62	62	67	67	68	63	66	67	68	53	63	60
CauMC4																							76	63	64	67	66	68	65	67	66	67	53	66	63
SacMC4																								66	64	70	68	70	66	68	68	68	54	64	60
HsaMC5																									80	78	70	72	69	69	70	70	52	62	63
MmuMC5																										79	69	70	71	69	69	71	53	60	60
GgaMC5																											74	77	72	76	75	76	53	65	62
TruMC5																												84	76	81	81	74	51	65	59
DreMC5a																													79	88	96	76	54	67	62
DreMC5b																														75	78	73	52	64	61
OmyMC5																															86	75	52	67	61
CauMC5																																76	54	66	62
SacMC5																																	55	66	61
LflMCa																																		52	54
LflMCb																																			65
MglMCc																																			
