# Supplementary figures and images for: Functional characterization of two melanocortin (MC) receptors in lamprey showing orthology to the MC1 and MC4 receptor subtypes
Source: BMC Evol Biol. 2007 Jun 29;7:101. doi: 10.1186/1471-2148-7-101 (PMC1925065; doi:10.1186/1471-2148-7-101)

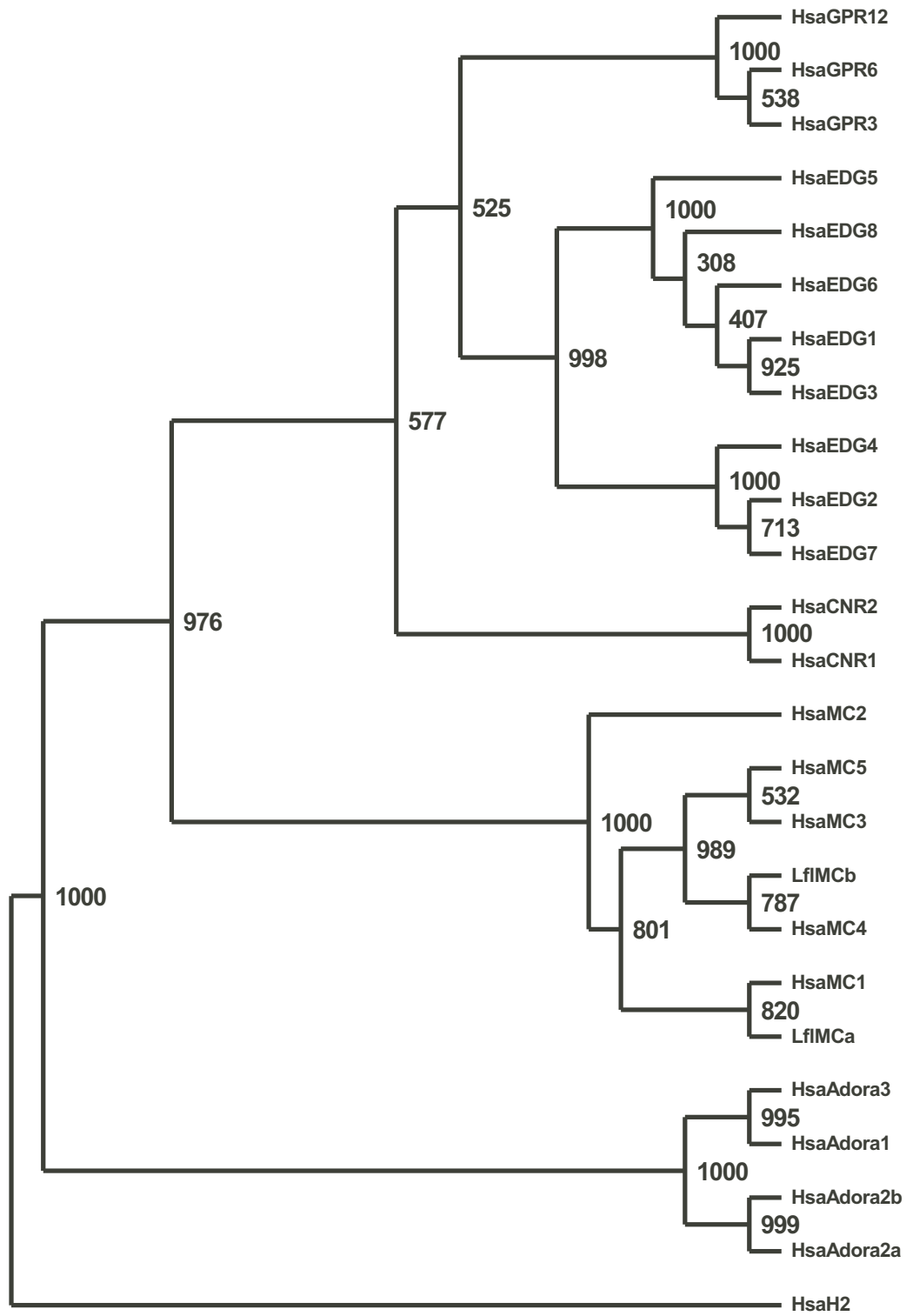

Supplement: Additional File 2 — Phylogenetic analysis of full-length amino acid sequences of lamprey MC receptors together with most closely related human α-group rhodopsin GPCRs. The consensus tree was generated by Neighbor-Joining analysis (Phylip 3.6a3). The numbers above the nodes indicate bootstrap replicates. The abbreviations used: Hsa, human and Lfl, lamprey. The sequences of annotated genes were downloaded from GenBank. [file 1471-2148-7-101-S2.pdf]

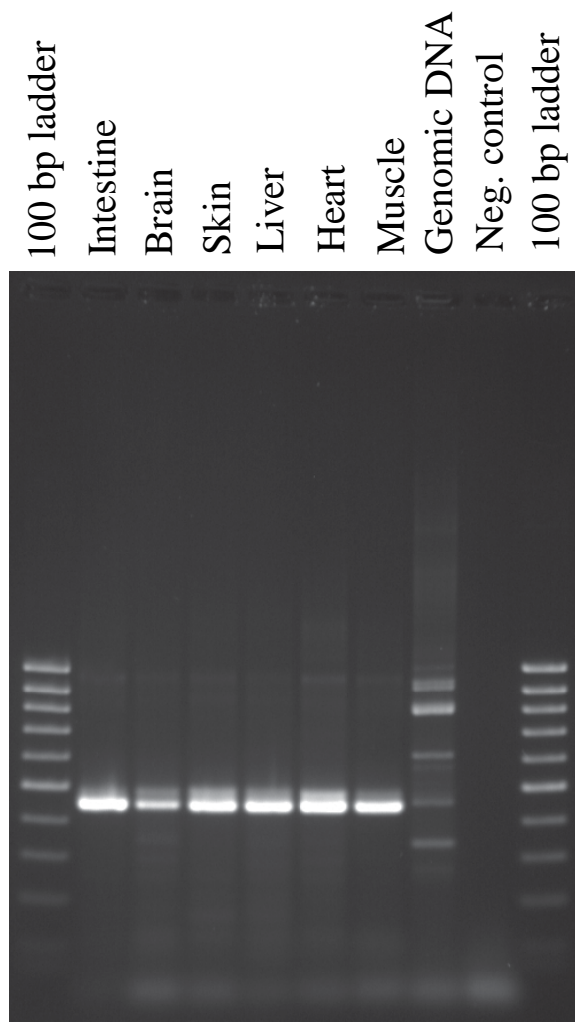

Supplement: Additional File 3 — Expression of lamprey β-actin mRNA, determined by RT-PCR. Expression is presented on ethidium bromide stained agarose gel. The tissues and controls are denoted at the top of the figure. The PCR was performed two times with qualitatively similar results. [file 1471-2148-7-101-S3.pdf]
